# Worsening of Primary Raynaud's Phenomenon During Episodes of Pyrexia and Rigors in SARS-CoV-2 Infection

**DOI:** 10.7759/cureus.33781

**Published:** 2023-01-14

**Authors:** Ciara J Bansal, Kirollos Kamel

**Affiliations:** 1 Rheumatology, Haematology, Royal Melbourne Hospital, Melbourne, AUS; 2 Haematology, Royal Melbourne Hospital, Melbourne, AUS

**Keywords:** sars-cov-2, pyrexia, digital ischemia, coronavirus, coronavirus disease, raynaud disease, raynaud phenomenon, mechanism, rigors, covid-19

## Abstract

Acute SARS-CoV-2 infection is associated with several cutaneous manifestations, including vasculitic digital ischemia. The reversal of digital ischemia in the primary Raynaud phenomenon (RP) arises from endothelial hypersensitivity to circulating adrenaline and noradrenaline and diminished vasodilatory innervation. SARS-CoV-2 can infect endothelial cells and be associated with raised adrenaline levels, reduced microvascular dilatory responses, and exaggerated clotting mechanisms. We report worsened RP in a patient with previously mild winter-time primary RP during the periods of fever and rigors from an acute SARS-CoV-2 infection contracted in a warm summer period. This reverted to the mild phenotype after recovery, and we discussed possible mechanisms for the brief exacerbation.

## Introduction

Primary Raynaud's phenomenon (RP) affects approximately 5% of the general adult population. The disorder comprises cold-induced episodic digital vasospasm, which in most people is primary and without an obvious cause [1]. However, several viral infections have been recorded to initiate RP, although this is not obviously related to fever or features of autoimmunity. Secondary RP is rarer and may arise in several medical conditions, with certain medications, after breast implant surgery [2], and in specific occupations [1].

It is common knowledge that SARS-CoV-2 infection is frequently accompanied by cardiorespiratory, neurological, and gastrointestinal symptoms. However, several types of skin rashes have also been reported. These include morbilliform and vesicular eruptions, petechiae, livedo reticularis-like rashes, and acro-ischaemic-like lesions such as lupus pernio (chill blains) [3 - 6]. In the latter, acral purpura with or without itching and discomfort lasting several days is evident [3]. In contrast to the reversible skin changes in RP, the more "fixed" skin rashes in SARS-CoV-2 are likely the result of endothelial damage induced by immune complexes and complement activation contributing to cellular stimulation and inflammatory cytokine release [6]. Importantly, new-onset RP has only rarely been reported following SARS-CoV-2 infection, but the latter can contribute to new-onset systemic sclerosis [7, 8] and aggravate secondary RP in established scleroderma [9].

The SARS-CoV-2 spike protein uses angiotensin-converting enzyme 2 as a receptor to gain entry into the human body and is known to infect endothelial cells [10]. In this respect, it can significantly reduce mean nailfold capillary numbers per linear millimeter in survivors of SARS-CoV-2 compared to patients with primary RP and healthy controls [11]. We report a case of mild and very infrequent primary RP that intensified during the fever and rigors of an acute SARS-CoV-2 infection during a warm summer spell and then settled back after recovery from the acute infection.

## Case presentation

**Figure 1:**
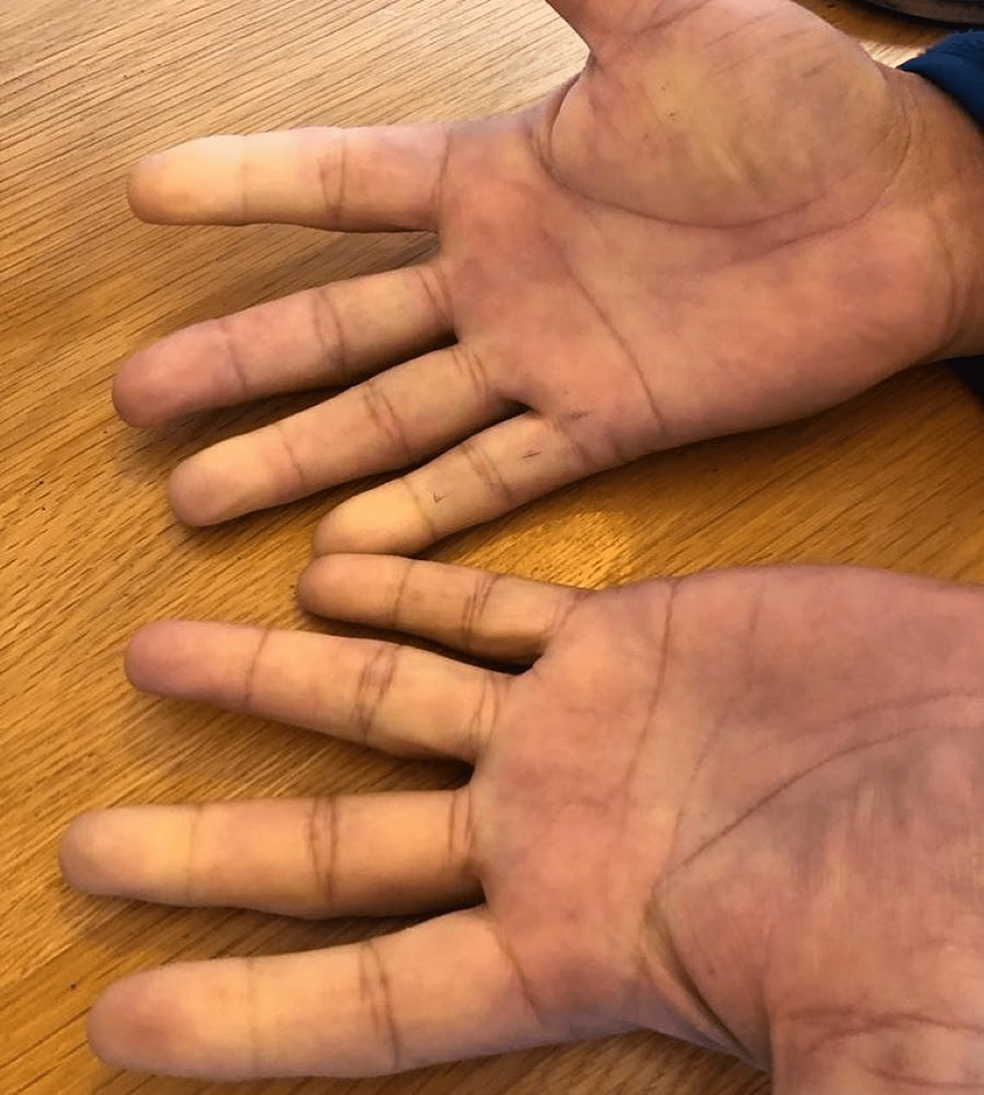
The patient showed a typical digital pallor during a bout of fever during an RP episode.

A 63-year-old lifelong non-smoking medical research doctor of Indian descent living in the UK had developed primary Raynaud's phenomenon (RP) in his fingers eight years earlier due to exposure to intense cold in the winter months. He had required a bioprosthetic aortic valve replacement four years later for idiopathic severe stenosis, and this was accompanied by a triple-vessel coronary artery bypass procedure. The RP had continued unchanged, despite the introduction of aspirin 75 mg, bisoprolol 2.5 mg, rosuvastatin 20 mg, and ramipril 2.5 mg daily. He had received two vaccinations with the Pfizer vaccine and then a booster over the preceding year, which had no impact on his RP. However, this reactivated significantly, causing several episodes of Raynaud's phenomenon daily for four days, when he developed repeated rigors during a highly positive lateral flow test (LFT) that confirmed the SARS-CoV-2 infection in the summer of 2022. The digital pallor was more extensive and prolonged (Figure 1) and occurred when the ambient temperature was often 21°C or higher and his oral temperature was equal to or above 38.9°C.

Physical examination showed normal skin without features of either lupus or scleroderma. Nailfold capillaroscopy was negative, and his blood pressure was satisfactory at 130/85 in both arms. He had normal peripheral pulses. Cardiac auscultation did not uncover any murmurs, and there were no crepitations suggestive of interstitial lung disease.

His routine tests showed a normal hemoglobin of 155 g/L, a white blood cell count of 7.8 x 109/L, and platelets of 345 x 109/L. The ESR was 4 mm and the CRP<1mg/dL, both of which were normal, and his electrolytes, urea, creatinine, liver enzymes, T4, and thyroid stimulating hormone were all normal. His antinuclear antibody (ANA) on HEp-2 cells was negative, as was his assessment for anti-Sjogren's syndrome (anti-SSA), Sjögren syndrome type B antigen (SS-B), scleroderma (Scl-70), ribonucleoproteins (RNP), anti-double-stranded deoxyribonucleic acid antibodies (dsDNA Abs), and Jo-1 (antihistidyl transfer RNA (t-RNA) synthetase) auto-antibodies. A recent chest X-ray did not disclose any infection, fibrosis, or cervical ribs.

As the patient’s infection and its associated headaches, sore throat, mild cough, and myalgia subsided over the ensuing nine days, so did his Raynaud phenomenon, which returned to its normal status. His LFT, checked two weeks after he became asymptomatic, was negative. The Omicron variant BA.5 was widely prevalent at the time our patient became infected. However, polymerase chain reaction (PCR) swabs and precise virus identification were not undertaken as there was no cardiorespiratory compromise, and he improved quite quickly. He remains well, with no significant RP except in very cold external temperatures or with the handling of frozen food.

## Discussion

In several infections, new-onset and often self-limiting RP has been reported. In the case of parvovirus B19 infection, immune-mediated endothelial damage is suggested to produce vasoconstriction and platelet activation [12]. In contrast, worsening of established RP has been reported in the case of cytomegalovirus infection in patients with underlying lupus [13]. The authors speculated on the ability of cytomegalovirus (CMV) to establish a particular pattern of vascular symptoms in those with lupus. More recently, SARS-CoV-2 has been shown to produce several types of vascular rashes, with acro-ischaemic findings occurring in 0.6% of outpatients and 2.9% of those admitted to an intensive care unit [3]. HIV and hepatitis C virus infection have also been linked to RP, especially in pediatric patients or those receiving chemotherapy or immune-based therapies [14, 15]. With respect to Helicobacter pylori (H. pylori) infection, initial reports suggested a higher prevalence in those with primary RP. However, Sulli et al. [16] showed no difference in positive 13C-urea breath tests in patients with primary versus secondary RP. More recently, Alak [17] found only one patient with a positive Helicobacter stool antigen test among the 29 patients with RP referred to a cardiovascular polyclinic.

The underlying mechanism leading to the digital ischemia of RP is likely multi-factorial and involves a combination of endothelial hypersensitivity to circulating adrenaline and noradrenaline and diminished vasodilatory innervation. RP in cutaneous vessels likely involves the cold-induced mitochondrial release of reactive oxygen species, which then induces activation of the Rho-Rho-kinase pathway. The latter in turn activates the noradrenaline-sensitive digital smooth muscle vasoconstrictor adrenaline a2C receptors [18]. In this respect, it is interesting that SARS-CoV-2 infection is associated with raised adrenaline levels and reduced microvascular dilatory responses [19] and that the microvascular changes persist following recovery from significant SARS-CoV-2 infection [11]. Furthermore, SARS-CoV-2 is known to induce depletion of endothelial ACE2, which can then lead to variably raised vasoconstrictor angiotensin 1 and reduced vasodilatory angiotensin 2 [20]. The increase in the frequency and intensity of the RP, in this case, maybe due to increased catecholamine release during the fever and rigors of the SARS-CoV-2 infection, similar to that seen in influenza infection [21] and other causes of elevated temperature [22]. Alterations in local angiotensin I and II modified by angiotensin-converting enzyme (ACE) inhibitor therapy are also likely to be important. It is unlikely that our patient developed systemic sclerosis, in which the RP has been linked to anti-cytokeratin 10 auto-antibodies [23] and increases in transforming factor beta, markers of coagulation, and endothelial damage [24]. Thus, he had no skin or lung signs of systemic sclerosis, and his RP settled back to its original intensity once he had recovered from the acute infection. However, temporary SARS-CoV-2 antibodies with low avidity that cross-react with adrenaline A2C receptors cannot be ruled out [25].

## Conclusions

SARS-CoV-2 can infect endothelial cells and be associated with reduced microvascular dilatory responses and raised adrenaline levels during periods of fever. These factors can worsen RP, which is due to increased vasoconstrictor sensitivity to noradrenaline and reduced vasodilatory innervation. Symptoms of RP are increased during periods of fever and rigors when adrenaline levels are specially raised but return to baseline on recovery from the acute infection.
